# The Effect of Zn Content on the Mechanical Properties of Mg-4Nd-xZn Alloys (x = 0, 3, 5 and 8 wt.%)

**DOI:** 10.3390/ma11071103

**Published:** 2018-06-28

**Authors:** Serge Gavras, Ricardo H. Buzolin, Tungky Subroto, Andreas Stark, Domonkos Tolnai

**Affiliations:** 1 Institute of Materials Research, Helmholtz-Zentrum Geesthacht, Max-Planck-Strasse 1, D 21502 Geesthacht, Germany; tungky.subroto@hzg.de (T.S.); andreas.stark@hzg.de (A.S.); domonkos.tolnai@hzg.de (D.T.); 2 Institute of Materials Science, Joining and Forming, Graz University of Technology, A8010 Graz, Austria; ricardo.buzolin@tugraz.at

**Keywords:** magnesium alloys, zinc addition, neodymium, Mg-Nd-Zn alloys, deformation behaviour, in situ synchrotron radiation diffraction

## Abstract

The mechanical properties of as-cast Mg-4Nd-xZn (x = 0, 3, 5 or 8 wt.%) alloys were investigated both in situ and ex situ in as-cast and solution-treated conditions. The additions of 3 or 5 wt.% Zn in the base Mg-4Nd alloy did not improve yield strength in comparison to the binary Mg-4Nd alloy. Mechanical properties were shown to improve only with the relatively high concentration of 8 wt.% Zn to Mg-4Nd. The change in intermetallic morphology from a continuous intermetallic to a lamella-like intermetallic was the primary reason for the decreased mechanical properties in Mg-4Nd-3Zn and Mg-4Nd-5Zn compared with Mg-4Nd and Mg-4Nd-8Zn. The dissolution of intermetallic at grain boundaries following heat treatment further indicated the importance of grain boundary reinforcement as shown in both in situ and ex situ compression testing. Azimuthal angle-time plots indicated little grain rotation most noticeably in Mg-4Nd, which also indicated the influence of a strong intermetallic network along the grain boundaries.

## 1. Introduction

The growing demand for increased efficiency in the transportation sectors has directed attention to lightweight solutions. Magnesium, as one of the lightest metals [1] was the focus of numerous pieces of research in recent years with the goal to overcome the effects of poor absolute mechanical properties and corrosion resistance [2]. This prior research lead not only to the improvement of the Mg alloys’ performance, but it was also shown that controlled corrosion allows for the use of certain Mg alloys as degradable implants in the medical sector [3].

Alloying Mg with Zn results in a combination of enhanced strength and ductility [4]. Owing to this performance, the alloy ZK60 (Mg-6.0Zn-0.6Zr (wt.%)) became one of the highest strength commercial wrought Mg alloy. Thus, the Mg-Zn alloys served as a foundation for the development of low cost Mg alloys and different elemental additions to this system has been investigated in order to develop the mechanical property profile through engineering of the grain boundary phases [5,6,7,8,9]. These investigations report further enhancement of elevated temperature strength and ductility [10,11] by the addition of Rare earth (RE) elements to the Mg-Zn-Zr system [12]. Neodymium having a relatively low solid solubility in Mg (3.6 wt.% at 549 °C [13]) is an ideal RE element because high concentrations are not needed to produce second phase particles in order to further improve the elevated temperature strength [14,15]. Furthermore, Nd is not toxic therefore the Mg-Nd-Zn system is under investigations for bio absorbable implant materials [16].

The evolution of synchrotron radiation based high energy X-ray and neutron diffraction advanced in situ characterisation methods [17,18]. The transmission geometry allows investigating bulk materials undergoing thermo-mechanical loading and to follow the dynamic microstructural processes occurring during processing [19]. The continuously acquired diffraction patterns and their evolution provide information on the grain structure and its changes, texture evolution, strain and strain anisotropy, which can be correlated with dislocation slip, twinning, recrystallization and recovery [20,21].

The tensile properties of as-cast Mg-4Nd and Mg-4Nd-8Zn at room temperature and 200 °C were investigated previously by Gavras et al. [22]. This current work builds on this by examining the influences of different Zn concentrations and solution treatment on Mg-Nd and Mg-Nd-Zn alloys.

## 2. Materials and Methods

A Mg-4Nd binary alloy was used as a base alloy and compared with ternary Mg-4Nd-xZn alloys with additions of 3, 5 and 8 wt.% of Zn in as-cast and solution treated conditions. The alloys were prepared by permanent mould indirect chill casting [23], an electric resistance furnace was used to melt the Mg under protective atmosphere of 2 vol.% SF_6_ and Ar. Zn and Nd were added as pure elements. After mixing, the melt was held at 720 °C for 10 min, then poured into a steel mould preheated to 660 °C. After holding at this temperature for 5 min, the mould was quenched in room temperature water at a rate of 10 mm s^−1^ until the top of the melt was in line with the cooling water level. Solution treatments were performed for 24 h at 525 °C for the Mg-4Nd, 480 °C for the Mg-4Nd-3Zn, 440 °C for the Mg-4Nd-5Nd and 420 °C for the Mg-4Nd-8Zn to prevent the partial melting of the intermetallics present in the different alloys.

For metallographic characterisation, as-cast and deformed samples were mounted in epoxy and ground using SiC paper and then polished using OPS solution. The microstructures for optical microscopy (OM) were etched with acetic-picral solution. The specimens for electron backscattered diffraction studies (EBSD) were not etched but washed immediately before the measurements with 0.5 vol.% nitric acid in ethanol solution for 5 s.

The optical microscopic analysis was performed using a Leica DMI 5000 light optical microscope (Wetzlar, Germany). Grain size measurements were obtained from optical micrographs using the line intercept method over an area of the sample covering approximately 100 grains. The scanning electron microscopy investigation was performed using a Tescan Vega3 SEM (Brno, Czech Republic) and a Zeiss FEG-SEM Ultra 55 (Oberkochen, Germany) attached with a Hikari detector (EDAX, Weiterstadt, Germany) and a TSL-OIM software (EDAX, Weiterstadt, Germany) package for Electron Backscatter Diffraction (EBSD) analysis. The EBSD measurements on compressed samples were conducted on an area of 700 μm × 700 μm with step size of 1 μm close to the centre of the deformed specimen to ensure a similar area to that measured during diffraction analysis. All microstructures of deformed samples are presented so that the compression direction is parallel to the horizontal direction.

The tensile and compression tests were performed on a Zwick Z050 universal testing machine (Zwick/Roell, Ulm, Germany). A maximum load of 50 kN was used to test samples at room temperature and at 200 °C, using a strain rate of 10^−3^·s^−1^. The tensile and compression tests were performed using the standard DIN 50125 [24]. with a minimum of five samples per condition and per alloy. Tensile and compression samples were cut from ingots using electron discharge machining (EDM). The dimensions of the tensile samples were 60 mm in total length with a gauge length of 35 mm and gauge diameters of 9.8 mm. The compression samples had a length of 15 mm with a diameter of 10 mm.

For the in situ compression experiments, cylindrical specimens were machined from cast ingots and solution treated specimens with a diameter of 5 mm and length of 10 mm. The in situ synchrotron radiation diffraction was performed at the P07 beamline of Petra III, DESY (Deutsches Elektronen-Synchrotron, Hamburg, Germany). A monochromatic beam with the energy of 100 keV (λ = 0.0124 nm) and with a cross section of 1 × 1 mm^2^ was used. Diffraction patterns were recorded with a PerkinElmer 1622 flat panel detector (Baesweiler, Germany) with a pixel size of (200 μm^2^) which was placed at a sample-to-detector distance of 1603 mm from the specimen (calibrated with a LaB_6_ standard powder sample). The acquisition time for each image was 1 s. The specimens were placed in the chamber of a dilatometer DIL 805A/D (TA Instruments, New Castle, DE, USA), combined with a modified heating induction coil in order for the beam to pass only through the sample [25]. The specimens were compressed at room temperature and at 200 °C. For the tests at 200 °C, the specimens were heated to the test temperature at a rate of 30 K·s^−1^ and held for 3 min before the compression started to ensure temperature homogeneity. The specimens were compressed with an initial strain rate of 1.0 × 10^−3^ s^−1^. The tests were terminated at a strain of 0.1. The morphology of the Debye-Scherrer rings was then analysed using the Fit2D^®^ software (ESRF, Grenoble, France) and converted into azimuthal-angle time (AT) plots by using the ImageJ^®^ software package (NIH, Bethesda, MA, USA).

## 3. Results

### 3.1. Metallography

The actual alloy compositions measured with spark analyser and X-ray fluorescence spectroscopy are listed in Table 1.

The microstructure and mechanical properties of Mg-4Nd, Mg-4Nd-3Zn, Mg-4Nd-5Zn and Mg-4Nd-8Zn were compared in as-cast and solution treated conditions. Solution treatments were chosen in order to get the highest concentrations of alloying additions into solid solution without causing the intermetallic phases present at grain boundaries to melt. The addition and then increasing concentration of Zn to the base Mg-4Nd alloys decreases the average grain size of the alloys (Table 2).

**Figure 1:**
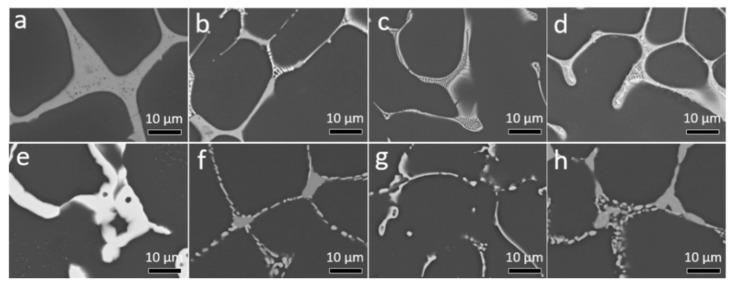
BSE SEM micrographs of as-cast (**a**) Mg-4Nd, (**b**) Mg-4Nd-3Zn, (**c**) Mg-4Nd-5Zn and (**d**) Mg-4Nd-8Zn and solution treated (**e**) Mg-4Nd (525 °C for 24 h), (**f**) Mg-4Nd-3Zn (480 °C for 24 h), (**g**) Mg-4Nd-5Zn (440 °C for 24 h) and (**h**) Mg-4Nd-8Zn (420 °C for 24 h).

The addition of Zn to the binary Mg-4Nd alloy results in a change to the morphology of the intermetallic phase present at grain boundaries. Figure 1 is used to illustrate the change in intermetallic morphology with increasing Zn additions. Due to the relatively high concentrations of Nd and Zn additions to Mg, not all of the intermetallic dissolved into solid solution following solution treatments.

### 3.2. Mechanical Properties

**Figure 2:**
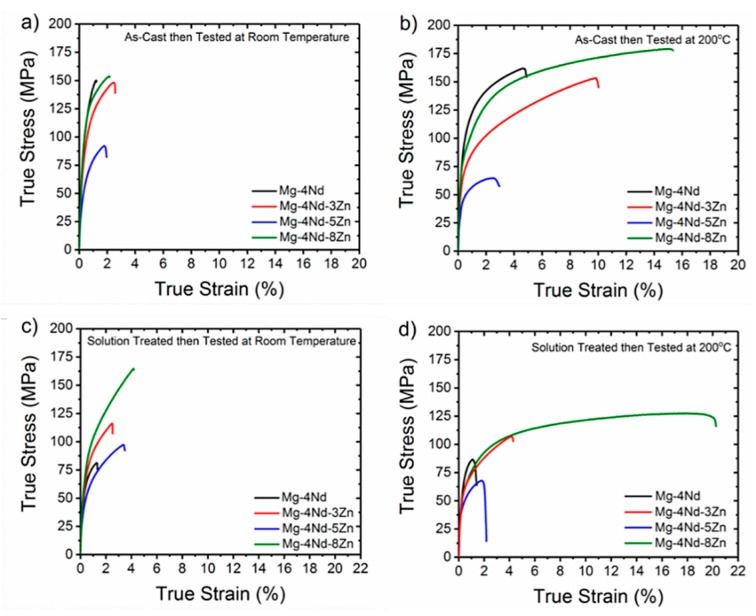
Representative tensile true stress—true strain curves for the as-cast and solution treated alloys in conditions (**a**) as-cast at room temperature, (**b**) as-cast at 200 °C, (**c**) solution treated at room temperature and (**d**) solution treated at 200 °C.

The binary Mg-4Nd alloy has superior tensile properties compared with the ternary Mg-4Nd-3Zn and Mg-4Nd-5Zn alloys (Figure 2). It is only when the relatively high concentration of 8 wt.% Zn is added to the Mg-4Nd that the ternary alloy properties improve. However, this is still not a significant improvement to tensile properties. In the as-cast condition, the addition of 8 wt.% Zn to Mg-4Nd, at best, only marginally improves the 0.2% proof stress (PS) and ultimate tensile strength (UTS) compared with Mg-4Nd. In the solution treated condition, however, the Mg-4Nd-8Zn performs significantly better than Mg-4Nd particularly with respect to the UTS (Table 3). The Mg-4Nd-5Zn alloy, in general, has significantly poorer 0.2% PS and UTS compared to the other alloys tested (Table 3). The elongation to failure properties are poor for all alloys in both as-cast and solution treated conditions. Elongation values do not exceed 5% at room temperature (Table 3). It is only at 200 °C when the Mg-4Nd-8Zn alloy in as-cast or solution treated conditions has an elongation to failure value greater than 10%.

Following solution treatment at 520 °C for 24 h the binary Mg-4Nd alloy has poorer room temperature and 200 °C tensile properties compared with both solution treated Mg-4Nd-3Zn (480 °C for 24 h) and Mg-4Nd-8Zn (420 °C for 24 h), Figure 2c,d. The Mg-4Nd-5Zn alloy continues to have the lowest 0.2% PS and UTS of all alloys tested (Table 3). It is important to note that solution treatment did not improve tensile properties for any of the alloys tested with exception to Mg-4Nd-8Zn.

**Figure 3:**
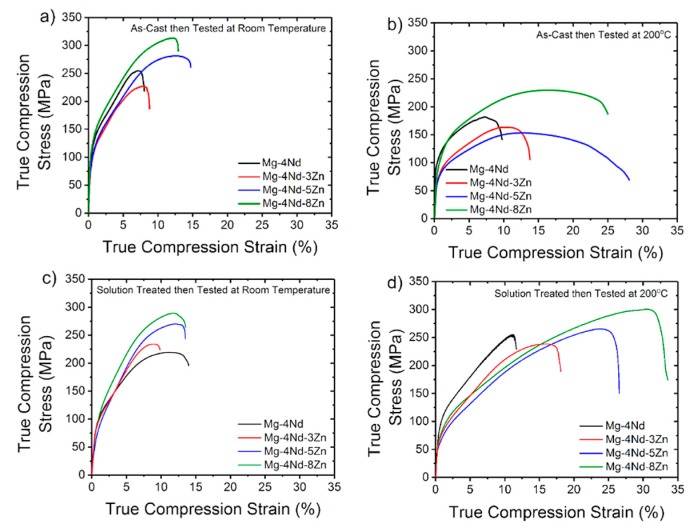
Compression true stress-strain curves for the as-cast and solution treated alloys in conditions (**a**) as-cast at room temperature, (**b**) as-cast at 200 °C, (**c**) solution treated at room temperature and (**d**) solution treated at 200 °C.

Under compression testing conditions, the alloys experienced approximately twice the amount of ultimate compressive strength (UCS) in comparison to UTS at room temperature in the as-cast condition (Table 4). The compression to failure experienced by the alloys is also greater than the elongation to fracture. The most notable change in the trend of property-to-alloying addition relation is that the Mg-4Nd-5Nd does not have the poorest compression properties in the as-cast condition at room temperature (Figure 3a). This is in contrast to the Mg-4Nd-5Zn alloy under tension at room temperature. There is no significant change to the alloy properties under compression at 200 °C in the as-cast condition to the room temperature compression properties. However, the binary Mg-4Nd alloy has the lowest UCS (Figure 3c) compared to the other alloys tested. This is once again different to the tensile properties of room temperature (RT) as-cast alloys.

Similar to the as-cast condition, the alloys tested at room temperature and 200 °C following solution treatment experience higher UCS than UTS. In both tension and compression at RT for as-cast and solution treated conditions, Mg-4Nd-8Zn has the highest 0.2% PS and Mg-4Nd-5Zn has the lowest 0.2% PS compared to the other alloys tested in the same conditions.

### 3.3. In Situ Compression Experiments

**Figure 4:**
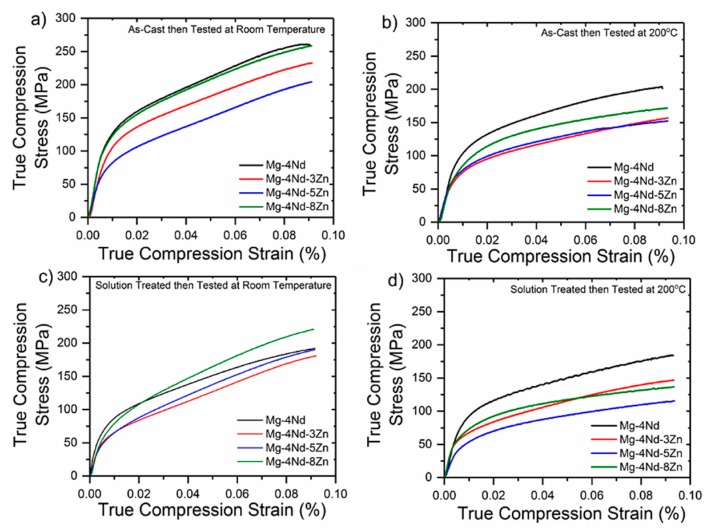
In situ compressive true stress-true strain curves for the as-cast and solution treated alloys in conditions (**a**) as-cast at room temperature, (**b**) as-cast at 200 °C, (**c**) solution treated at room temperature and (**d**) solution treated at 200 °C.

The true stress-true strain curves from the in situ compression tests are shown in Figure 4. Regarding the as-cast alloys tested at room temperature, a decrease in the maximum compressive stress was observed with the addition of Zn for the Mg-4Nd-3Zn and Mg-4Nd-5Zn. For the Mg-4Nd-8Zn the proof and maximum compressive stresses are comparable with the Mg-4Nd. For the tests performed at 200 °C, the Mg-4Nd exhibited the highest proof stress, followed by Mg-4Nd-8Zn. Mg-4Nd-5Zn and Mg-4Nd-3Zn exhibited comparable proof stresses. The maximum compressive stress was exhibited by Mg-4Nd-8Zn.

The in situ compression tests at room temperature of the T4 specimens show that Mg-4Nd exhibited the highest proof and maximum compression stresses, followed by the Mg-4Nd-8Zn (Table 5). The Mg-4Nd-3Zn and Mg-4Nd-5Zn alloys exhibited the lowest values. At 200 °C, the Mg-4Nd alloy also exhibited the highest proof and maximum compression stresses. The decrease in the compressive strength comparing to room temperature and 200 °C was more significant for the Mg-4Nd-5Zn, which exhibited the lowest proof and maximum compressive stresses at 200 °C. In both in situ and ex situ compression investigations the Mg-4Nd-5Zn alloy is shown to have the lowest 0.2% PS in comparison to the other alloys tested.

**Figure 5:**
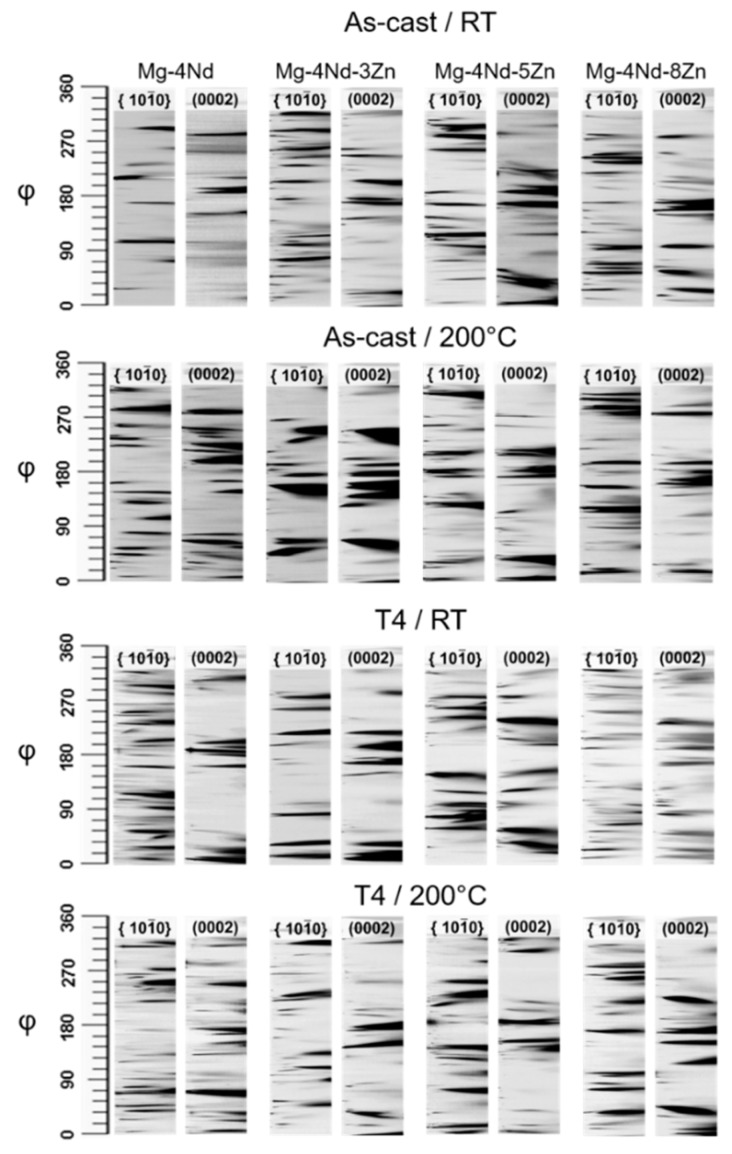
Azimuthal-angle time plots (AT) plots obtained during the in situ synchrotron radiation diffraction during compressive deformation at room temperature and 200 °C.

Figure 5 shows the Azimuthal-angle time plots (AT)-plots for the as-cast alloys. Regarding the as-cast alloys, the AT-plots of the alloy compressed at 200 °C, the bending of the timelines was slightly more pronounced than at room temperature. Nevertheless, the bending of the timelines was very low for all alloys up to 10% compression, either at room temperature or 200 °C. The small grain rotation suggests that the microstructure is very stable, which can be explained by the presence of a rigid network of intermetallic compounds along the grain boundaries. The broadening of the timelines was more pronounced at 200 °C compared with room temperature, indicating that subgrain formation has occurred during deformation at this temperature. The as-cast alloy that exhibited the most stable microstructure, i.e., negligible grain rotation and not significant subgrain formation was the Mg-4Nd alloy. Discrete spots in the AT-plots were not observed after the beginning of deformation for any as-cast alloy neither deformed at room temperature nor at 200 °C. This indicates that discontinuous dynamic recrystallization did not play any role on the compression mechanisms of the investigated alloys.

Similar to the results for the as-cast alloys, the heat treated Mg-Nd-Zn alloys did not exhibit significant microstructural changes that are indicated in the AT-plots. The appearance of new timelines at the early stages of deformation is apparent for all alloys. The Mg-4Nd is the only alloy that also shows the appearance of timelines at late stages of deformation, especially for the $\left(10\bar{1}0\right)$ plane, suggesting intensive twinning during compression. Although a number of new spots in the AT-plots of the Mg-4Nd alloy deformed at 200 °C are visible, no strong evidence that discontinuous dynamic recrystallization played an important role on the deformation of the Mg-4Nd is observed in the microstructure.

**Figure 6:**
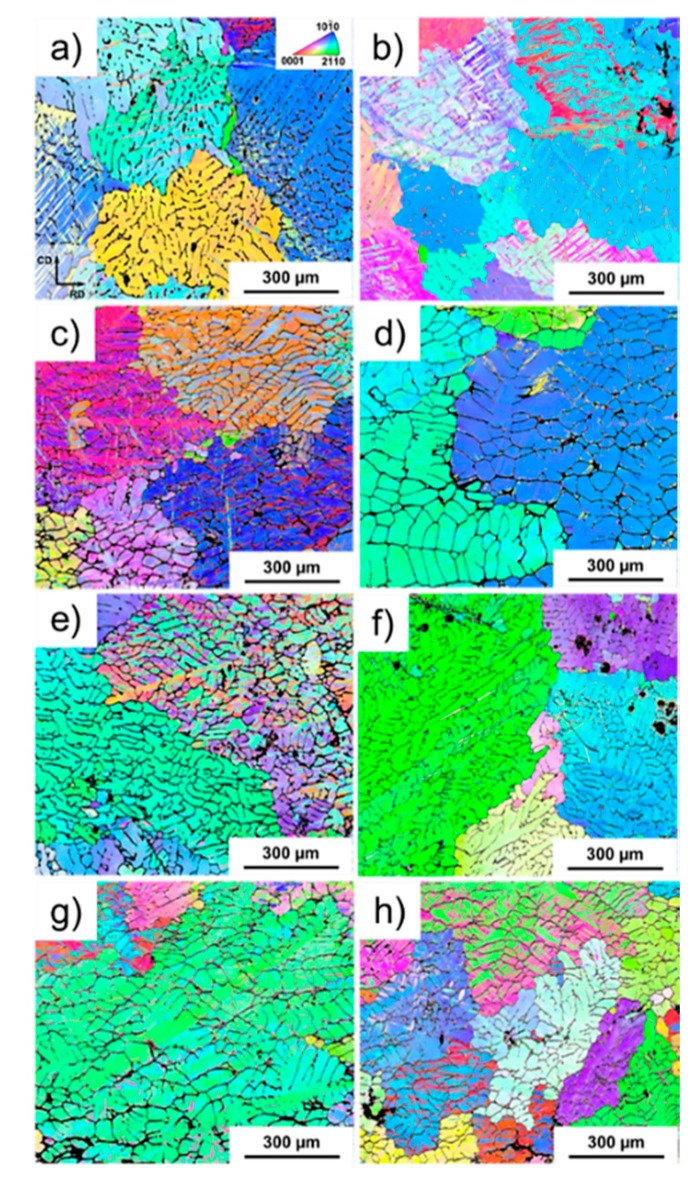
EBSD inverse pole figure maps of as-cast samples subjected to compressive strain at RT (**a**,**c**,**e**,**g**) and at 200 °C (**b**,**d**,**f**,**h**) of Mg-4Nd (**a**,**b**), Mg-4Nd-3Zn (**c**,**d**), Mg-4Nd-5Zn (**e**,**f**) and Mg-4Nd-8Zn (**g**,**h**).

**Figure 7:**
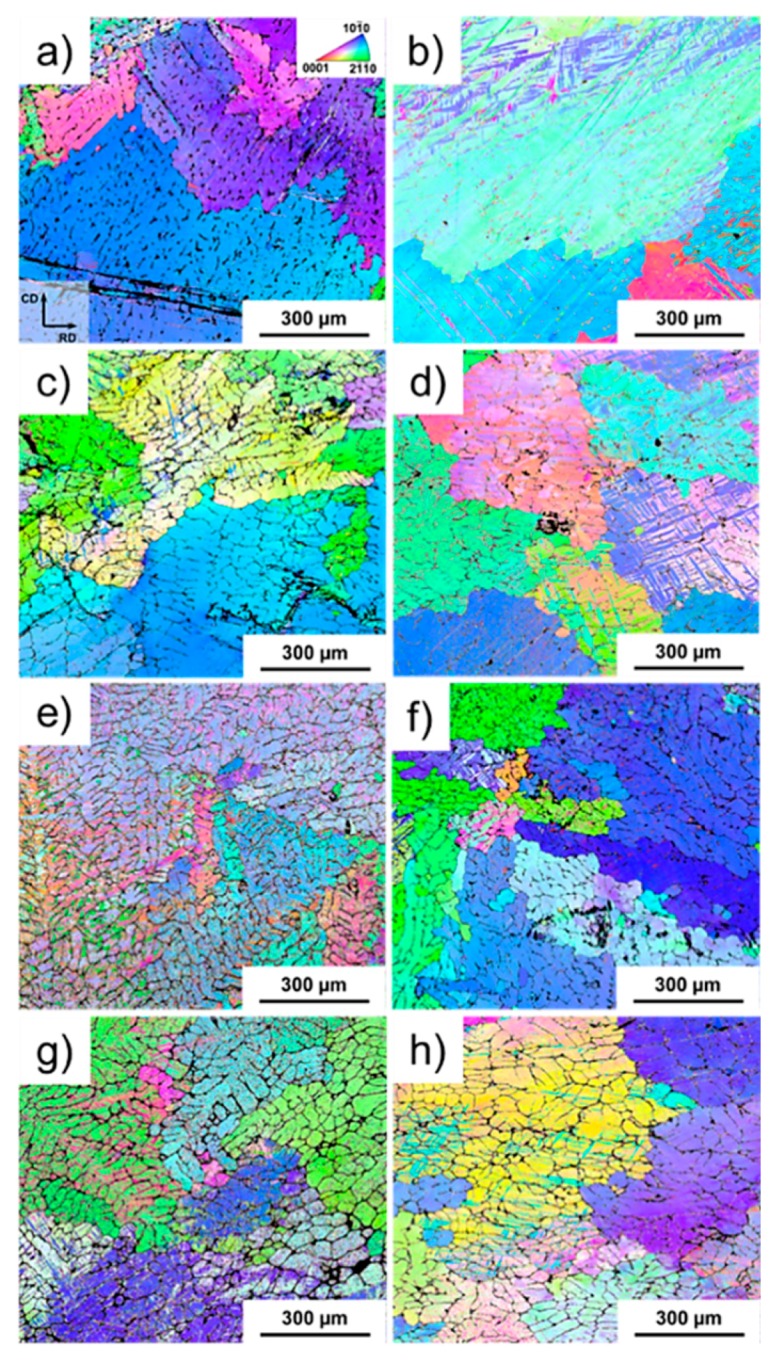
EBSD inverse pole figure maps of solution treated samples subjected to compressive strain at RT (**a**,**c**,**e**,**g**) and at 200 °C (**b**,**d**,**f**,**h**) of Mg-4Nd (**a**,**b**), Mg-4Nd-3Zn (**c**,**d**), Mg-4Nd-5Zn (**e**,**f**) and Mg-4Nd-8Zn (**g**,**h**).

Figure 6 shows the IPF maps of the alloys for the as-cast alloys in situ compressed at room temperature Figure 6a,c,e,g and at 200 °C Figure 6b,d,f,h. For the alloys deformed at room temperature, fine twins are present along the grains that were in favourable orientation for twinning. A similar microstructure is observed for Mg-4Nd-3Zn, with slightly higher volume fraction of twins in comparison with Mg-4Nd. Mg-4Nd-5Zn alloy also shows a notably twinned microstructure. The thickness of the twins seems to be larger for the Mg-4Nd-5Zn, which suggests that there were regions with significant stress concentration during deformation in this alloy. The misorientation spread for the solution treated alloys compressed at 200 °C was significantly smaller compared with the alloys compressed at room temperature (Figure 7), suggesting that dynamic recovery played an important role on the deformation of the Mg-Nd-Zn alloys at this temperature. No evidence of recrystallised grains originating from discontinuous dynamic recrystallization was found. For Mg-4Nd some twins could be observed. Please define, if appropriate.

## 4. Discussion

As shown previously, the intermetallic phase in the binary Mg-4Nd is Mg_12_Nd [26]. The addition of 3, 5 and 8 wt.% Zn to the base Mg-4Nd alloy results in two distinct intermetallic morphologies being present at grain boundaries, Mg_50_Nd_8_Zn_42_ and Mg_3_(Nd, Zn) [22].

It is not immediately apparent as to why the Mg-4Nd-5Zn alloy generally performed more poorly than any of the other alloys investigated in this currently work. However, by investigation of the microstructure it can been seen that the intermetallic morphology of the Mg-4Nd is broad and continuous at grain boundaries (Figure 1a). The intermetallic morphology of Mg-4Nd-5Zn differs to that of Mg-4Nd (Figure 1). The Mg-4Nd-5Zn alloy has a less continuous and more lamellar-like appearance. The morphologies of the intermetallic present in Mg-4Nd-3Zn and Mg-4Nd-8Zn are more similar to that of Mg-4Nd-5Zn. This indicates that the continuous intermetallic phase (Mg_12_Nd) has a significant strengthening effect on the Mg-4Nd alloy which was similarly shown in Mg-Nd and Mg-La-Nd alloys by Zhang et al. [27]. Particularly since the volume fraction of the Mg_12_Nd phase in Mg-4Nd is less than that of the intermetallic in the ternary alloys investigated (Table 2). However, with sufficient addition of Zn (namely Mg-4Nd-8Zn) the tensile and compressive properties of Mg-Nd-Zn is improved.

The dissolution of intermetallics at grain boundaries following solution treatment further indicates the importance of a strengthening intermetallic to obtain superior mechanical properties. The general reduction of 0.2% PS and ultimate tensile and compression strength of the solution treated alloys in comparison to as-cast alloys is also related to the reduction of grain boundary reinforcement. This indicates that although the intermetallic morphology present in the ternary alloys appears to be less able to withstand tensile or compressive loads, once a sufficient amount of grain boundary reinforcement is present, tensile or compressive properties improve.

In the AT plots each timeline corresponds to a coherent domain that is in Bragg position and therefore the diffraction occurs. In this case, when the timeline is a single line that indicates that the misorientation spread within the coherent domain is negligible [19]. A cast or recrystallized microstructure will exhibit discrete timelines. When the material starts to deform the microstructure must accommodate the strain. Geometrical conditions will give the most favourable deformation mechanism: basal glide or twinning. The disappearance of a timeline in the $\left(10\bar{1}0\right)$ plane and the subsequent appearance in the (0002) in the AT-plots indicates twinning. It is also known that with the increase of temperature, prismatic or pyramidal slipping can also be activated. At temperatures normally higher than 150 °C dynamic recovery and recrystallization also play an important role on the deformation of magnesium. Among the possible phenomena to be observed in an AT-plot, subgrain formation also plays an important role on the deformation and is indicated by the blurring of a timeline. The spreading of the coherent domains indicates that there is an increase in misorientation, thus formation of cells and subsequently subgrains form. The diffraction results and the post experiment grain structure of the alloys shows that twinning was the predominant deformation mechanism at room temperature for all alloys in as-cast condition (Figure 6). Mg-4Nd exhibited an intensive twinned microstructure, as well as the Mg-4Nd-3Zn alloy. The twins seem thicker for the Mg-4Nd-3Zn. Twinned grains are also visible in Mg-4Nd-5Zn, but a few grains did not exhibit twins. For the Mg-4Nd-8Zn, the twins are significantly thicker compared with Mg-4Nd. The graphs of the alloys deformed at 200 °C reveal that twins are still present in most of the deformed microstructure of the alloys, although the area fraction seems to reduce substantially for Mg-4Nd-3Zn and especially Mg-4Nd-5Zn, in which twins are barely observed. Nevertheless, intensive subgrain formation indicated by the gradual change of colour within the grain is observed for the alloys compressed at 200 °C and is notable for the Mg-4Nd-5Zn alloy.

The alloys in solution treated condition (Figure 7) exhibit local twinning with regions that were subjected to higher levels of localised stresses. This indicates that the strain is not homogeneously accommodated by the microstructure. This suggests that the reinforcement of the matrix was not effective to redistribute homogeneously the stresses during deformation. Grains were partially twinned for the Mg-4Nd alloy. At 200 °C intensive subgrain formation can be observed for all the alloys, more pronounced in the case of Zn containing ones. A few twins can be also seen, but these tend to be thicker, and also fully twinned regions can be observed.

## 5. Conclusions

The Mg-4Nd alloy has superior mechanical properties to the ternary alloys. The change in intermetallic morphology from a continuous intermetallic to a lamella-like intermetallic is the primary cause of the reduction in mechanical properties. Only the addition of an excessive amount of Zn (8 wt.%) reaches the properties of the base alloy. The UCS is twice as large as the UTS value tested at RT in as-cast condition. The ternary alloys do not perform better after solution treatment. The deformation at RT happens mostly through twinning, at 200 °C subgrain formation occurs. There is no notable grain rotation observed in the microstructure of the as-cast alloys indicating strong grain boundary strengthening from the intermetallic. In the case of solution treated samples the microstructure undergoes twinning in a local manner. This localization can be a result of the insufficient load distribution of the intermetallic particles as the heat treatment disintegrates its network.

## Figures and Tables

**Table 1:** Chemical compositions of the alloys.

Alloy (wt.%)	Nd wt.% (XRF)	Zn wt.% (Spark Analyzer)
Mg-4Nd	4.20	–
Mg-4Nd-3Zn	4.35	3.20
Mg-4Nd-5Zn	4.20	5.20
Mg-4Nd-8Zn	4.34	8.00

**Table 2:** Average grain size of as-cast and solution treated alloys.

Alloy (wt.%)	Grain Size in As-Cast Condition (mm) ± SD	Grain Size in Solution Treated Condition (mm) ± SD
Mg-4Nd	0.99 ± 0.14	1.13 ± 0.07
Mg-4Nd-3Zn	0.55 ± 0.03	0.76 ± 0.04
Mg-4Nd-5Zn	0.36 ± 0.02	0.38 ± 0.01
Mg-4Nd-8Zn	0.20 ± 0.02	0.14 ± 0.02

**Table 3:** Ex situ tensile properties of alloys tested at room temperature or 200 °C in as-cast or solution treated conditions.

Alloy (wt.%)	Ave 0.2% PS ± SD (MPa)	Ave UTS ± SD (MPa)	Ave Elong ± SD (%)
**As-Cast Tested at Room Temperature**			
Mg-4Nd	103.4 ± 1.8	147.7 ± 17.0	1.2 ± 0.3
Mg-4Nd-3Zn	81.7 ± 2.1	143.6 ± 6.6	2.5 ± 0.3
Mg-4Nd-5Zn	50.0 ± 1.5	84.9 ± 10.1	1.7 ± 0.7
Mg-4Nd-8Zn	105.1 ± 1.9	151.4 ± 2.1	2.0 ± 0.1
**Solution Treated Tested at Room Temperature**			
Mg-4Nd	53.6 ± 4.5	80.2 ± 28.5	2.0 ± 1.0
Mg-4Nd-3Zn	66.7 ± 11.7	113.0 ± 20.1	2.5 ± 0.7
Mg-4Nd-5Zn	46.1 ± 10.9	107.7 ± 31.1	3.2 ± 0.7
Mg-4Nd-8Zn	71.1 ± 3.1	163.9 ± 27.7	4.3 ± 1.7
**As-Cast Tested at 200 °C**			
Mg-4Nd	96.8 ± 2.1	169.3 ± 12.1	4.9 ± 1.3
Mg-4Nd-3Zn	69.7 ± 2.7	152.8 ± 3.4	9.9 ± 1.4
Mg-4Nd-5Zn	44.9 ± 1.0	66.1 ± 3.2	3.2 ± 0.8
Mg-4Nd-8Zn	83.9 ± 3.2	177.7 ± 4.7	15.2 ± 0.5
**Solution Treated Tested at 200 °C**			
Mg-4Nd	68.5 ± 15.5	83.4 ± 4.7	1.6 ± 0.3
Mg-4Nd-3Zn	62.3 ± 9.5	97.1 ± 8.6	3.7 ± 1.0
Mg-4Nd-5Zn	43.8 ± 1.3	66.9 ± 3.1	1.9 ± 0.4
Mg-4Nd-8Zn	61.4 ± 1.3	125.1 ± 2.6	17.8 ± 6.8

**Table 4:** Ex-situ compression properties of alloys tested at room temperature or 200 °C in as-cast or solution treated conditions.

Alloy (wt.%)	Ave 0.2% PS ± SD (MPa)	Ave UCS ± SD (MPa)	Ave Comp. ± SD (%)
**As-Cast Tested at Room Temperature**			
Mg-4Nd	103.2 (±5.0)	257.4 (±11.3)	8.3 (±0.7)
Mg-4Nd-3Zn	87.9 (±2.8)	226.3 (±6.7)	9.0 (±0.9)
Mg-4Nd-5Zn	88.9 (±1.3)	283.1 (±5.0)	13.7 (±1.0)
Mg-4Nd-8Zn	115.0 (±1.6)	311.9 (±4.4)	13.0 (±1.7)
**Solution Treated Tested at Room Temperature**			
Mg-4Nd	87.1 (±2.4)	285.0 (±20.6)	14.4 (±2.1)
Mg-4Nd-3Zn	80.9 (±2.4)	285.1 (±6.2)	11.3 (±1.2)
Mg-4Nd-5Zn	69.0 (±5.1)	356.6 (±14.1)	13.9 (±1.2)
Mg-4Nd-8Zn	78.9 (±3.7)	375.4 (±3.7)	13.2 (±0.2)
**As-Cast Tested at 200 °C**			
Mg-4Nd	99.5 (±7.1)	198.8 (±13.1)	10.2 (±1.0)
Mg-4Nd-3Zn	68.8 (±3.2)	203.7 (±10.9)	12.3 (±1.1)
Mg-4Nd-5Zn	67.8 (±1.9)	218.7 (±9.4)	25.8 (±2.1)
Mg-4Nd-8Zn	89.7 (±0.4)	230.1 (±0.3)	26.4 (±1.0)
**Solution Treated Tested at 200 °C**			
Mg-4Nd	87.3 (±4.2)	258.3 (±5.5)	12.4 (±0.6)
Mg-4Nd-3Zn	67.2 (±1.4)	238.6 (±5.5)	18.0 (±2.0)
Mg-4Nd-5Zn	55.8 (±3.4)	265.9 (±8.6)	27.8 (±2.0)
Mg-4Nd-8Zn	69.3 (±0.5)	300.2 (±9.8)	33.9 (±1.5)

**Table 5:** In situ compression 0.2% proof stress of alloys tested at room temperature or 200 °C in as-cast or solution treated conditions.

0.2% Proof Stress ± SD (MPa)				
Alloy (wt.%)	As-Cast at RT	As-Cast at 200 °C	Solution Treated at RT	Solution Treated at 200 °C
Mg-4Nd	106.8 (± 5.2)	80.4 (± 3.8)	63.2 (±1.5)	74.4 (±1.4)
Mg-4Nd-3Zn	88.2 (±2.9)	66.9 (±1.6)	56.2 (±2.9)	50.5 (±3.3)
Mg-4Nd-5Zn	69.2 (±1.5)	66.7 (±1.5)	50.2 (±1.4)	48.3 (±2.4)
Mg-4Nd-8Zn	100.3 (±4.4)	83.4 (±8.0)	63.5 (±0.1)	57.7 (±1.6)
